# ForCenS-LGM: a dataset of planktonic foraminifera species assemblage composition for the Last Glacial Maximum

**DOI:** 10.1038/s41597-024-03166-7

**Published:** 2024-04-10

**Authors:** Lukas Jonkers, Alan Mix, Antje Voelker, Bjørg Risebrobakken, Christopher W. Smart, Elena Ivanova, Elsa Arellano-Torres, Frédérique Eynaud, Haddam Naoufel, Lars Max, Linda Rossignol, Margit H. Simon, Maria Virgínia Alves Martins, Sandro Petró, Thibaut Caley, Trond Dokken, Will Howard, Michal Kucera

**Affiliations:** 1 MARUM Center for Marine Environmental Sciences, University of Bremen, 28359 Bremen, Germany; 2 College of Earth, Ocean, and Atmospheric Sciences, Oregon State University, Corvallis, OR 97331-5503 USA; 3 Instituto Português do Mar e da Atmosfera (IPMA), Divisão de Geologia e Georecursos Marinhos. Av. Doutor Alfredo Magalhães Ramalho, 6, 1495-165 Alges, Portugal; 4 Centro de Ciências do Mar do Algarve (CCMAR), Universidade do Algarve, 8005-139 Faro, Portugal; 5 NORCE Norwegian Research Centre, Bjerknes Centre for Climate Change, Jahnebakken 5. NO-5007, Bergen, Norway; 6 School of Geography, Earth and Environmental Sciences, University of Plymouth, Drake Circus, Plymouth PL4 8AA UK; 7 Shirshov Institute of Oceanology, Russian Academy of Sciences, Moscow, Russia; 8 Escuela Nacional de Ciencias de la Tierra (ENCiT), Universidad Nacional Autónoma de México, Mexico City, Mexico; 9 Univ. Bordeaux, CNRS, Bordeaux INP, EPOC, UMR 5805, F-33600 Pessac, France; 10 GEOPS Géosciences Paris‐Sud, CNRS, Université de Paris Sud Paris Saclay, Orsay, Cedex France; 11 LSCE/IPSL Laboratoire des Sciences du Climat et de l’Environnement, CEA‐CNRS‐UVSQ, Orme des Merisiers, Saint‐Aubin, France; 12 Universidade do Estado do Rio de Janeiro, UERJ, Faculdade de Geologia, Av. São Francisco Xavier 24, Lab. 4037F, Maracanã, 20550-013 Rio de Janeiro Brazil; 13 Universidade de Aveiro, GeoBioTec, Departamento de Geociências, Campus de Santiago, 3810-193 Aveiro, Portugal; 14 itt OCEANEON - Instituto Tecnológico de Paleoceanografia e Mudanças Climáticas, UNISINOS - Universidade do Vale do Rio dos Sinos, São Leopoldo, Brazil; 15 Fenner School of Environment & Society, Australian National University, Canberra, ACT Australia

**Keywords:** Palaeoceanography, Biogeography

## Abstract

Species assemblage composition of marine microfossils offers the possibility to investigate ecological and climatological change on time scales inaccessible using conventional observations. Planktonic foraminifera - calcareous zooplankton - have an excellent fossil record and are used extensively in palaeoecology and palaeoceanography. During the Last Glacial Maximum (LGM; 19,000 – 23,000 years ago), the climate was in a radically different state. This period is therefore a key target to investigate climate and biodiversity under different conditions than today. Studying LGM climate and ecosystems indeed has a long history, yet the most recent global synthesis of planktonic foraminifera assemblage composition is now nearly two decades old. Here we present the ForCenS-LGM dataset with 2,365 species assemblage samples collected using standardised methods and with harmonised taxonomy. The data originate from marine sediments from 664 sites and present a more than 50% increase in coverage compared to previous work. The taxonomy is compatible with the most recent global core top dataset, enabling direct investigation of temporal changes in foraminifera biogeography and facilitating seawater temperature reconstructions.

## Background and Summary

Palaeoecological data holds the unique promise to provide insights into ecosystem and climate change on time scales that are inaccessible through direct observations^1–4^. Planktonic foraminifera are marine zooplankton that are globally ubiquitous in the near-surface ocean^5^. They build calcite shells, which upon death of the organisms, sink and, are, under the right conditions preserved in seafloor sediments. Calcareous sediments with abundant and well-preserved planktonic foraminifera are globally distributed at water depths above ~4000 metres. The species composition of their sedimentary assemblages presents a vertically and temporally integrated reflection of the living species community^6^. The group has an exceptional fossil record that stretches back to the Jurassic^7,8^. Because the species composition of planktonic foraminifera assemblages is sensitive to temperature^9,10^, fossil assemblages not only offer a chance to study ecosystem and biogeography changes^11–14^, but also provide a basis for quantitative reconstructions of seawater temperature by means of a range of (multivariate) approaches that relate species composition to seawater temperature^15,16^. In fact, one of the first global compilations of fossil planktonic foraminifera assemblages was conducted for this purpose^17^.

This first compilation within the CLIMAP (Climate: Long range Investigation, Mapping, and Prediction) project focussed on the period known as the Last Glacial Maximum (LGM), marked by the largest ice sheets in the recent past, to obtain a reconstruction of global temperature from a climate state fundamentally different from today. An important aim of CLIMAP was to provide temperature reconstructions that could be used both as boundary conditions and tests for climate models. Since then, new syntheses of LGM planktonic foraminifera assemblages were built^18^ and the most recent, global compilation was carried out within the MARGO (Multiproxy Approach for the Reconstruction of the Glacial Ocean) project^16,19^. The data have been used extensively for global temperature reconstructions^20,21^ and for comparison with climate model simulations^19,22,23^. In addition, the data have provided a window into a marine ecosystem under markedly different climate conditions and prior to human influence that is unaffected by the appearance or extinction of species^11,24^.

Lingering uncertainty about the state of the last glacial ocean^25,26^, new developments in transfer function modelling^27,28^, novel ways to analyse palaeoecological data and the prospect to use such data to evaluate climate models directly, demonstrate the relevance of syntheses of planktonic foraminifera assemblages. The long history of using planktonic foraminifera assemblage data for quantitative palaeoecology has also ensured fairly well-standardised data collection protocols, rendering synthesis of such data relatively straightforward. Almost two decades have passed since the MARGO foraminifera dataset for the LGM was published and valuable new data has become available. Here we present an update to the MARGO compilation, the ForCenS-LGM dataset, that presents an extension in data coverage by more than 50%, especially in the North Atlantic. We anticipate that this extended LGM planktonic foraminifera assemblage dataset will be of use to palaeoceanographers and palaeoecologists alike.

## Methods

Since the ForCenS-LGM dataset presents an update to the MARGO dataset, we follow the same inclusion criteria. That means that we use the same temporal definition of the LGM: 19,000–23,000 years BP^29^ and follow their system of ranking of chronological confidence^30^. In practice, this means that samples with at least two calibrated ^14^C dates within the LGM interval are assigned chronozone level 1. Chronozone level 2 indicates samples that are bracketed by at least two radiometric dates within the 12–30 ka BP interval, or have age control based on correlation to time series with level 1 age control using for instance benthic foraminifera oxygen isotope ratios. Level 3 is similar to the hypothesis-based age control as specified for level 2, but with correlations targets that themselves have chronozone level 2. Level 4 is used to indicate sites without chronozone assignment and does not indicate sites with low chronological confidence. We use the age-depth models as provided in the publication describing the data. Even though these criteria were outlined in the MARGO project, their application and archiving was not consistently followed, resulting in a large number of sites with chronozone level 4. However, subsequent sensitivity tests showed negligible influence of chronological confidence on the reconstructions, testifying to the stability of the LGM and the temporally smoothed nature of the sedimentary record.

For practical reasons of data availability, we have not added calendar ages to data included in previous syntheses of LGM planktonic foraminifera assemblage composition. For other, new, data, we include the chronology as published in the paper describing the assemblage data. The reliance on the published chronologies is primarily because, for most sediment cores, age control information (radiocarbon ages, foraminifera stable isotope data, etc) is not (publicly) available, thus preventing reevaluation of the published age-depth models. However, since this dataset is intended to provide plankton assemblage composition representative of the four thousand year long LGM period and not to provide information on the temporal evolution of assemblage composition through time, we think using the original chronology is not critical. For the purposes of this dataset, stratigraphic consistency and applicability are likely to be of more immediate importance than absolute (calendar) chronology. The latter may evolve with new information such as improved radiocarbon calibrations. Users interested in analysing the precise temporal evolution of marine plankton species composition might want to use other resources^31,32^.

We only include assemblages with complete taxonomic coverage and counts made on specimens >150 μm. The latter also ensures compatibility with the ForCenS coretop dataset^33^, which can be used as a modern counterpart to this LGM compilation, as well as for transfer function calibration.

We have searched relevant literature and public data repositories (PANGAEA, National Center for Environmental Information) for new planktonic foraminifera assemblage data. In most cases this was done by searching for foraminifera-based temperature reconstructions using the following terms “plank* AND foram* AND assemblage” or “plank* AND foram* AND transfer function”. Virtually all additions stem from sedimentary time series of planktonic foraminifera assemblages, from which we selected the relevant samples^34–83^. Our update includes all data from a previous synthesis^84,85^ that was only partially included in MARGO and which was re-checked for chronological confidence. When available, data was assembled from public sources. Alternatively, data was sourced from publications or obtained from the authors of the publications in case it was not publicly available. The data compiled here was originally generated by manually identifying and counting the species present using a (binocular) microscope. Counts were in general based on aliquots of the sample containing at least 300 specimens as previous work has shown that this number is sufficient to capture the biodiversity^86^. In some cases (n = 108, of which 52 from two cores), particularly in low diversity assemblages, data from counts of fewer than 300 specimens were included. Whenever possible, specimen counts were archived. In most other cases the data is available as relative abundances. To further increase spatial coverage, we include previously unpublished count data from a single core with known chronology^87^. The assemblage data for the eight samples were generated using the methods described above.

Relevant metadata was compiled for all samples and is included in the data file. No attempt was made to fill out missing metadata for data included in the MARGO synthesis, but some minor corrections have been made. These were predominantly corrections to the position or water depth of the sites and are indicated whenever applicable. We have however updated the publication information associated with the MARGO data to allow users to cite individual data points.

Despite the largely standardised sampling protocols, individual researchers tend to use different taxonomies, thus necessitating taxonomic standardisation in order to compare assemblages from different sources. To a large degree, taxonomies differ in the names of the species and differences can hence be resolved through synonym mapping. Some taxonomies contain more species, but such splitting of species is rarer, but can be resolved through lumping. The opposite, taxonomies with fewer species, is fortunately rare, except for recently described (sub)species. Despite recent developments in planktonic foraminifera taxonomy^88,89^, we chose to follow the same taxonomy as ForCenS^33^. This choice ensures compatibility with the latter dataset, but also arises from practical issues as most legacy datasets do not resolve all currently known or accepted species. Changes to the taxonomy (renaming and lumping) are documented in the data file. Species not indicated in the original counts were assumed to be absent. This is a reasonable assumption and common practice in the field^33^.

## Data Records

The ForCenS-LGM dataset^90^ is available at PANGAEA using the following link: 10.1594/PANGAEA.962852. An earlier version of the dataset with fewer samples and less thorough checking of the chronological confidence is available on Zenodo^91^ to ensure reproducibility of other work^83^. We recommend using the dataset described here.

### Format

The format of the ForCenS-LGM dataset is a wide comma-separated text file. Each row contains the metadata and the species abundance data for a single sample. Species abundances are represented either as fractions (percentages) or as raw counts and in rare cases as accumulation rates. Many sites contain information from multiple samples within the LGM time frame.

### Column description

Each site is described using a core name (**Core** in the data file), coring device (**CoreDevice**), position (**Longitude,** **Latitude**), water depth (**WaterDepth_m**) and Ocean basin (**Ocean**). Of these, core name and position are considered essential metadata and available for all sites. The ocean basin designation follows earlier work^16^ and is intended for the application of regional transfer functions. Researchers can use the position data for alternative spatial grouping of the data.

Samples are accompanied by an indication of their depth within the sediment core (**SampleDepthTop_m**; **SampleDepthBottom_m**; **SampleDepthMid_m** of which at least one is considered essential) and an optional sample label (**SampleLabel**). Chronological information is contained in optional columns for age and sedimentation rate (**CalendarAge_cal_ky_BP**; **SedimentationRate_cm_ky**, respectively) and an indication of the chronological confidence following the criteria described above (**ChronozoneLevel**). The laboratory where the analyses were carried out is indicated in the column **Laboratory**. **Publication** contains information about where the data have been described first. Where possible, this information is provided using a digital object identifier. The column **ChronologySource** is only relevant to the new counts presented here and documents the origin of the chronological information used to define the LGM time interval. When available, a link to the data source is indicated in the column **Source**. For completeness the file also lists the date of addition (**DateOfAddition**) and the name of the person who added the data (**AddedBy**). Notes on the taxonomic harmonisation are included in **TaxonomicNotes** and other quality control issues are mentioned in **QCNote**. The file further contains a column **Count** indicating the total number of shells counted. This column can be used to screen for samples with low count sums. We made no attempt to convert relative abundances to absolute abundance in cases where the count sum is available as this is unlikely to yield only integers because of rounding errors that have accumulated over time. Finally, the file contains four columns that are included for practical purposes. **siteID** presents a simplified identifier for each site and **sampleID** for each sample; **isCounts** and **inMARGO** are boolean variables indicating whether or not the data is presented as counts (rather than relative abundances) and if the sample was part of the MARGO compilation, respectively. These are primarily included to allow filtering of the data. All other columns contain species abundances. Genus and species names are separated using an underscore.

## Technical Validation

The ForCenS-LGM dataset contains planktonic foraminifera abundance data from 41 species and 3 (sub)species groups (*Globorotalia menardii* and *G. tumida*, *Globigerinoides ruber ruber* (*G. ruber* according to the ForCenS taxonomy) and *Globigerinoides ruber alba* (designated informally as *G. white*), *Turborotalita humilis* and *Berggrenia pumilio*). When available, information on the coiling direction of *Globorotalia truncatulinoides* and *Turborotalita quinqueloba* is indicated, as is the abundance of specimens with and without a final sac-like chamber in *Trilobatus sacculifer*.

**Fig. 1:**
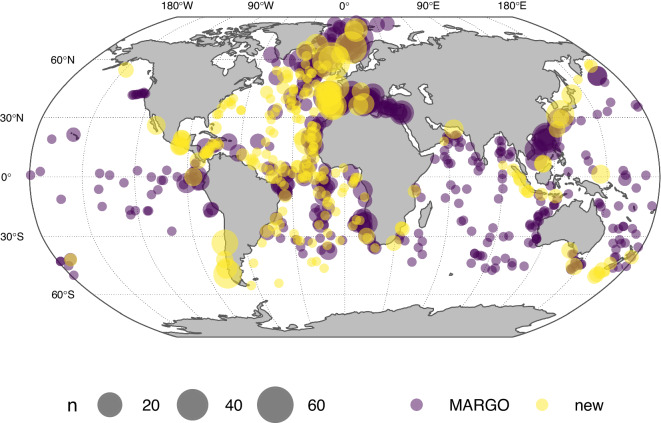
Distribution of sites in the ForCenS-LGM compilation. Symbol size is scaled to the number of samples per site and the colour indicates new additions since MARGO^16^. The ForCenS-LGM compilation expands the coverage by over 50%, with a particularly large increase in the number of data points in the Atlantic Ocean.

**Fig. 2:**
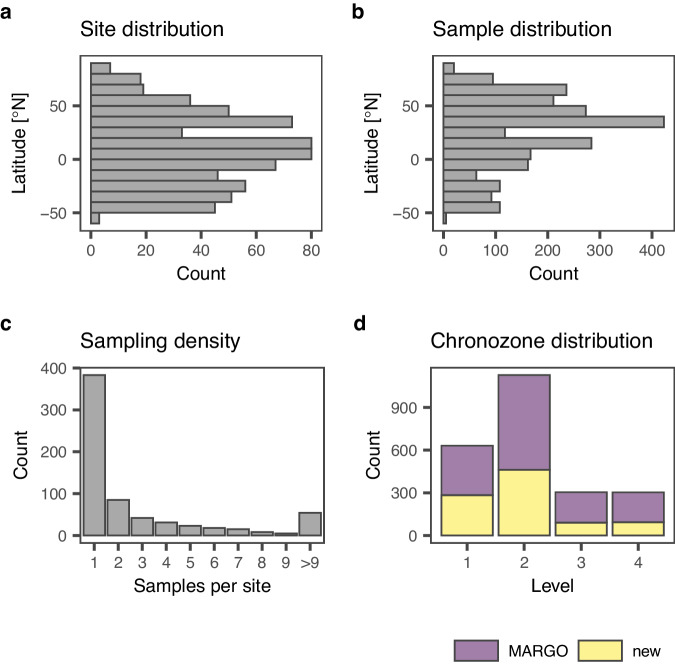
Key characteristics of the ForCenS-LGM dataset: latitudinal distribution of sites (**a**) and samples (**b**), number of samples per site (**c**) and chronozone level per sample (**d**). Note that chronozone level 4 does not reflect low chronological confidence, but is assigned to sites where the chronology was not, or could no longer be, evaluated following the criteria specified for the MARGO project.

The dataset contains data from 2,365 samples from 664 unique sites, compared to 1,437 and 428 in MARGO, respectively (Fig. 1). Most new sites are in the Atlantic basin, with a marked increase in coverage in the low to mid-latitude North Atlantic. Coverage in the Pacific and in the Southern Ocean remains poor, in part because of logistical issues with retrieving samples from these remote areas, but also because of issues associated with preservation of calcium carbonate in these deep basins. The majority of the sites is characterised by a single sample, but over 40% of the sites have at least two samples, offering the chance to assess within LGM variability to some degree at a large number of sites (Fig. 2). The chronology of approximately three quarters of the sites has been evaluated and the majority has been assigned to chronozone level 2.

**Fig. 3:**
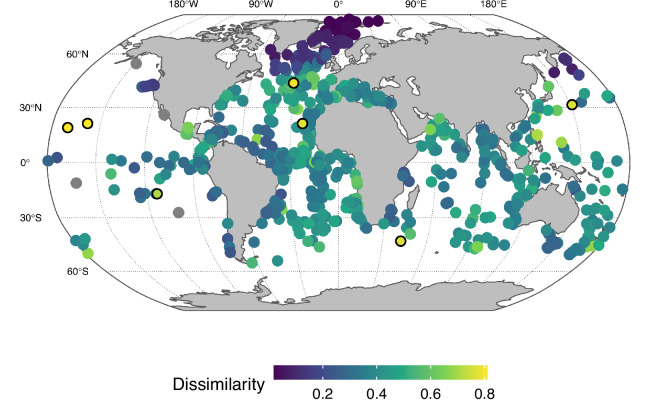
Spatial heterogeneity in the species assemblages in the ForCenS-LGM compilation. Colours show the average Bray-Curtis dissimilarity to all assemblages within a 1,500 km radius (grey dots are sites without neighbours within this radius). In general, this analysis demonstrates the spatial consistency of the data set, despite the various sources of uncertainty. To a first order, spatial consistency shows a link with species diversity (Fig. 4), showing larger heterogeneity in areas with higher species diversity. This approach also indicates a way to detect spurious assemblages. For instance, sites with assemblages that fall outside the 99^th^ percentile of the observed dissimilarities are indicated with a black circle.

**Fig. 4:**
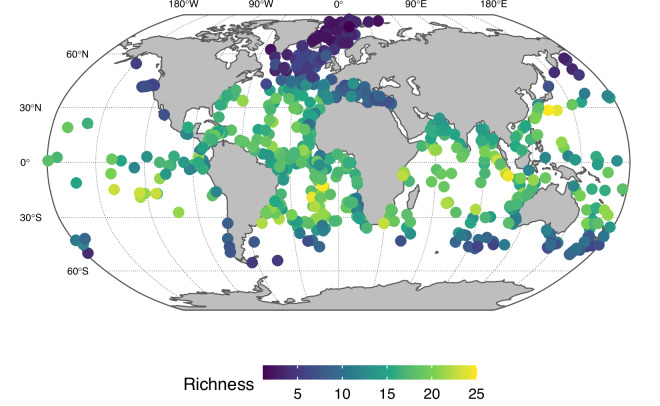
Average species richness for all sites in the ForCenS-LGM compilation. Like today, the latitudinal diversity gradient, with high biodiversity in the tropics and low diversity near the poles, was present during the LGM. This visualisation also highlights the spatial consistency of the compilation, despite the wide range of data sources.

The spatial variability among the LGM planktonic foraminifera assemblages can in theory be inflated due to post-depositional alteration, analytical and chronological uncertainty and archiving errors. One way to assess the influence of these factors is to investigate the spatial consistency of the compilation. We do this here by calculating for each site the average dissimilarity to all samples within a 1,500 km radius (Fig. 3). To a first order, this analysis reveals a link with species diversity, showing smaller heterogeneity in colder areas with lower species diversity (Fig. 4). This illustrates the challenges associated with the consistent characterisation of high-diversity species assemblages. The approach also highlights a few samples with unexpectedly high dissimilarity. In most cases these samples stem from areas with low spatial coverage, such as the Pacific Ocean indicating situations where the encountered assemblage composition represents a key enrichment of the dataset. In other cases, such as in the North Atlantic, the observed dissimilarity is unexpected and may warrant closer inspection depending on research needs. However, in general, the data set shows high spatial consistency. This indicates that even though the species counts were made by dozens of different researchers and over a time frame spanning decades and in the presence of uncertainty about the chronology and the degree to which the samples provide an accurate representation of the entire 4,000 year period, the compilation can be used to obtain a reasonable picture of the distribution of planktonic foraminifera during the LGM. Indeed, the ForCenS-LGM compilation shows clear biogeographical patterns, such as the latitudinal diversity gradient (Fig. 4), demonstrating the utility of the dataset for studying long-term changes in plankton biogeography, quantitative palaeoecology and palaeoceanography on basin and global spatial scales.

## Usage Notes

Species counts at ten sites in the Mediterranean, at 19 sites in the Pacific and one in the South Atlantic are not fully resolved. At these sites, white and pink subspecies of *G. ruber* are not distinguished in the Mediterranean and South Atlantic and *Globorotalia menardii* and *G. tumida* are lumped in the Pacific. Researchers will need to lump the species from the remaining sites if they deem it important to include those sites in their analysis. Lumping may be done on an ocean basin basis for the development of transfer function models as the effect of endemism or of cryptic species with different ecology has been shown to be reduced when regional transfer functions are used^16^.

The dataset contains the necessary data for further quality control if these are needed for analyses. Assemblages from great water depths may be influenced by dissolution, rendering resistant species overrepresented in the assemblages^92^. The user can explore the effect, and if needed filter the data, using the water depth of each site. In addition, assemblages are likely to be affected by bioturbation, potentially mixing assemblages of species that never co-occurred. This effect is likely to be more pronounced at low accumulation rates and the user may want to filter out assemblages from sites with low sediment accumulation rates. Not all samples are accompanied by an estimate of sedimentation rates. In these cases we suggest that a sediment depth threshold (e.g. 40 cm) may be used instead, as the depth of the LGM in any given core is a function of sedimentation rate.

The ForCenS-LGM compilation contains species assemblage data expressed as counts (integers), as relative abundances (either fractions or percentages) and in rare cases in other ways (accumulation rates, shells per unit weight of sediment). No correction has been attempted for assemblages where the relative abundances did not sum to unity, as it proved often impossible to trace the origin of the error. However, in most cases the uncertainty is small and likely the result of rounding. If desired, the user can filter out assemblages that deviate too much. For most research needs, the data will need to be standardised to account for differences in sampling effort and sediment accumulation rates. However, we explicitly refrain from doing so here in order to preserve the count data as they allow for quality control and rarefaction analysis. With the count data available, the user can also combine counts from different samples from the same site if the count numbers appear insufficient to capture the biodiversity. A final reason to preserve the count data is that conversion to relative abundances is prone to lead to downstream errors as a result of rounding and (future) changes in the taxonomy. With this in mind, we also recommend that researchers archive their microfossil abundance data using clear and complete taxonomy and report species counts, rather than relative abundances.

With automated processing of sediment samples and species identification still in its infancy, acquiring fossil assemblage data remains a manual and laborious effort. From the median number of specimens counted per sample, we estimate that this synthesis is based on over 870,000 specimens. Their identification requires specialist taxonomic knowledge and often involves manual manipulation of single specimens measuring a few 100s of a μm. Hence, to acknowledge the work needed to generate this data, we encourage users of the dataset to also cite the original data source whenever possible.

## Data Availability

No custom code was used to process the data.
